# BRASH Syndrome Presenting With Idioventricular Escape Rhythm in a Patient With Trifascicular Block

**DOI:** 10.7759/cureus.32217

**Published:** 2022-12-05

**Authors:** Henry O Aiwuyo, Nosakhare P Ilerhunmwuwa, Narek Hakobyan, Ephrem Sedeta, Ifeanyi Uche, Mustafa Wasifuddin, Beatrice E Torere, Jamal C Perry, Shahrokh E Rafii

**Affiliations:** 1 Internal Medicine, Brookdale University Hospital Medical Center, Brooklyn, USA; 2 Internal Medicine, Saba University School of Medicine, Brooklyn, USA; 3 Internal Medicine, North Mississippi Medical Center, Tupelo, USA; 4 Medicine, California Institute of Behavioral Neurosciences & Psychology, Fairfield, USA; 5 Cardiology, Brookdale University Hospital Medical Center, Brooklyn, USA

**Keywords:** av nodal disease, renal failure, bradycardia, hyperkalemia, brash syndrome

## Abstract

Bradycardia, renal failure, atrioventricular (AV) nodal disease, shock, and hyperkalemia (BRASH) syndrome is a well-recognized constellation of distinct clinicopathologic entities comprising bradycardia, renal failure, AV nodal disease, shock, and hyperkalemia. Our patient is an 89-year-old female with a past medical history significant for hypertension and diabetes, who was newly started on labetalol and had recent gastroenteritis; she presented to our Emergency Department with bradycardia and shock. Upon presentation, she showed physical signs of volume depletion, and her blood pressure was 50 mmHg systolic and heart rate was 25 beats per minute. The initial electrocardiogram showed an idioventricular rhythm. The laboratory workup revealed hyperkalemia. The patient was given repeated doses of atropine with no significant response. She was resuscitated with isotonic fluids. The patient improved clinically, her blood pressure stabilized, her potassium level, renal function, and heart rate were normalized, and normal sinus rhythm was restored with a narrow QRS complex. A diagnosis of BRASH syndrome was made retrospectively. Overall, the treatment of this syndrome is largely symptomatic. Hemodynamic support with fluid and treatment of hyperkalemia remains the goal of care. The overall prognosis is good if identified early and managed appropriately.

## Introduction

A constellation of bradycardia, renal failure, atrioventricular (AV) nodal disease, shock, and hyperkalemia (BRASH) is represented in this entity called BRASH syndrome. Since 2016 when it was first described by Farkas [1], there have been well-documented occurrences of this phenomenon in medical literature; however, little is known regarding its epidemiology. Case reports indicate a higher prevalence among elderly individuals. Some studies have described it as a primary metabolic disturbance in the form of hyperkalemia leading to a vicious cycle of bradycardia and shock, while others observed it mainly in the setting of a primary electrical disturbance of the heart precipitated by AV nodal blockade leading to hemodynamic alterations. As an emerging concept, details of its various presentations and optimal management protocols are yet to be completely understood. We present a case of an elderly woman who presented with this syndrome having a background history of cardiac conduction system disease.

## Case presentation

Our patient is an 89-year-old female who was brought to the Emergency Department (ED) after losing consciousness. She was found by the emergency medical services (EMS) to be hypotensive and bradycardic. According to her healthcare assistant, she had a history of nausea and vomiting for about three days prior to the collapse and was also noticed to be dehydrated. She was recently placed on labetalol for an unclear indication, prior to the onset of symptoms. Upon EMS arrival, her heart rate was 25 beats per minute (bpm) and 0.5 milligrams (mg) of atropine were immediately administered. In the ED, her heart rate was 36 bpm and her systolic blood pressure was 50 mmHg. Her home medications included hydralazine, isosorbide dinitrate, labetalol, trazodone, Lexapro, calcium, magnesium oxide, amlodipine, and clonidine. On physical examination, the patient had altered mental status with no respiratory distress and no significant findings on systemic examination. Admitting investigations are presented in Table 1.

**Table 1:** Findings from investigations at admission ↑, above normal range; ↔, within normal range; ↓, below normal range; WBC, white blood cell; BUN, blood urea nitrogen; ALT, alanine transaminase; AST, aspartate transaminase

Test	Finding	Reference range
Hemoglobin (g/dL)	10.5 ↓	11.4 – 15.5
Platelets (uL^-1^)	270 ↔	180 – 400
WBC (uL^-1^)	9.3 ↔	4.2 – 10.2
Serum potassium (mmol/L)	5.8 ↑	3.5 – 5.1
Magnesium (mg/dL)	1.4 ↓	1.6 – 2.3
Troponin (ng/mL)	0.9 ↑	< 0.034
Lactate (mmol/L)	4.00 ↑	0.7 – 2.1
D-dimer (ng/mL)	2,100 ↑	< 230
BUN (mg/dL)	38 ↑	7 – 17
Creatinine (mg/dL)	1.68 ↑	0.52 – 1.04
Urine sodium (mmol/L)	49 ↔	30 – 90
Ferritin (ng/mL)	296 ↑	11.4 – 264
ALT (u/L)	44 ↑	< 35
AST (u/L)	43 ↑	14 – 36

**Figure 1:**
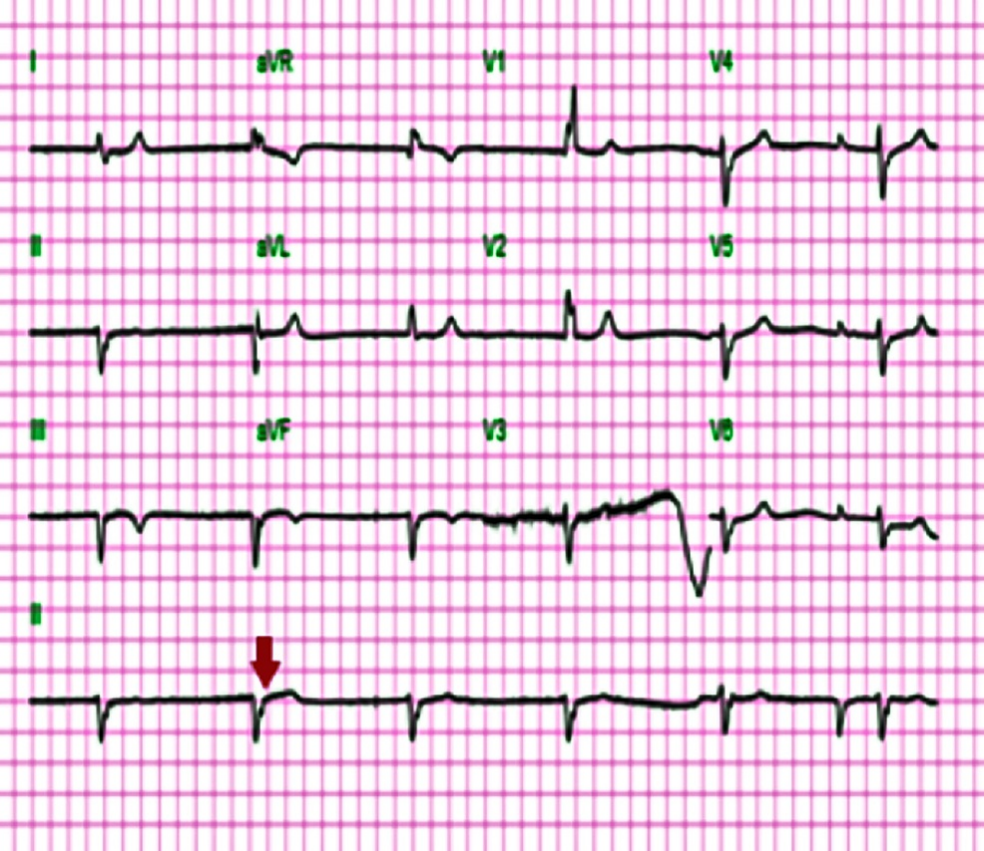
Idioventricular rhythm Admitting ECG (arrow) shows widened QRS complex

**Figure 2:**
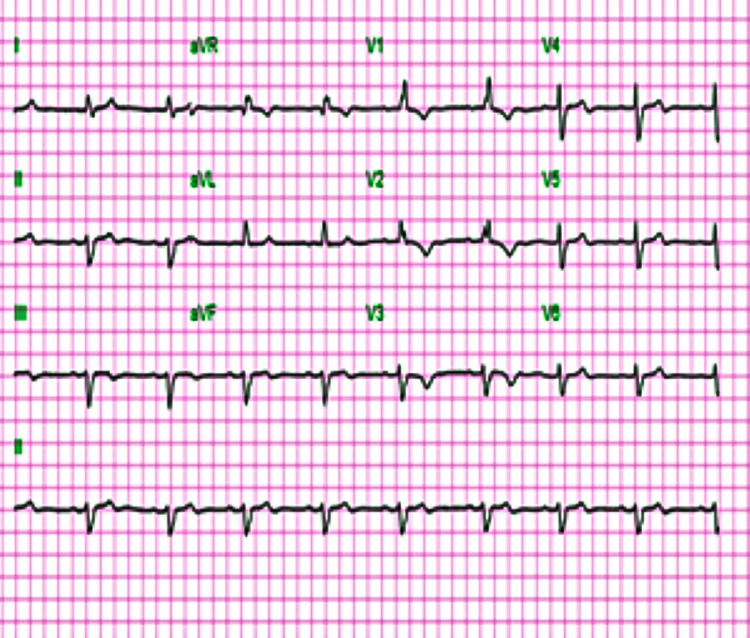
ECG post-treatment

An initial electrocardiogram (ECG) revealed an idioventricular rhythm (Figure 1). She received 2-liter bolus of intravenous normal saline and a further dose of atropine. Despite adequate dosing with atropine, she remained hemodynamically unstable. A transcutaneous pacemaker was applied but it was not utilized. Her vital signs improved subsequently, and she was admitted to the Coronary Care Unit (CCU) for further management. Her repeat ECG after treatment showed a normal sinus rhythm, first-degree AV block, right bundle branch block, and left anterior fascicular block, which was similar to her pre-admission ECG (Figure 2).

**Figure 3:**
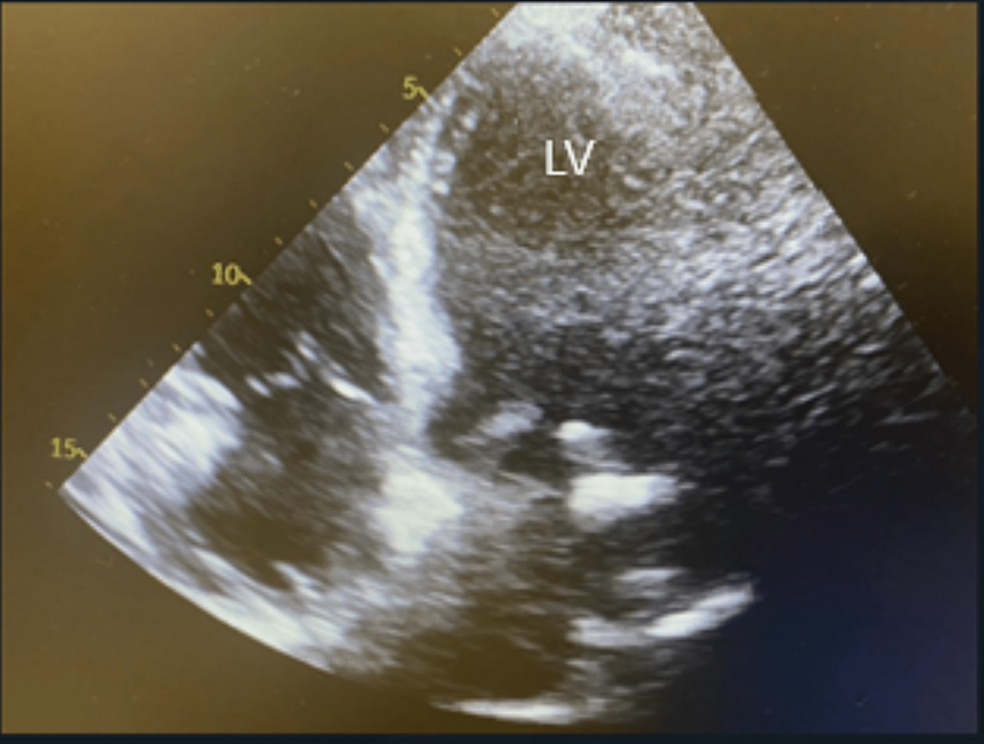
Two-dimensional echocardiography (four-chamber long-axis view) LV, left ventricle

Echocardiography revealed a normal left ventricular systolic function (Figure 3). There were no regional wall motion abnormalities. Trans-mitral Doppler revealed a pseudo-normal left ventricular filling pattern, suggesting increased filling pressure. The right atrium and ventricle were dilated, and right-sided systolic function was reduced with a pulmonary artery pressure of 50 mmHg. There was mildly calcific aortic sclerosis.

She underwent nuclear stress imaging, which demonstrated a left ventricular ejection fraction estimated to be 64% and fixed inferior wall infarction with no evidence of stress-induced ischemia. With regard to the inferior infarction management, no management was required as the patient already had a fixed infarction and there was no evidence of ongoing stress-induced ischemia on nuclear stress testing. A ventilation-perfusion scan showed a low probability for pulmonary embolism. Chest X-ray revealed mild cardiomegaly. Over the course of the admission, her potassium levels and renal function normalized, and her hemodynamics improved significantly. She was discharged from the CCU and advised to avoid AV nodal blocking agents.

## Discussion

**Figure 4:**
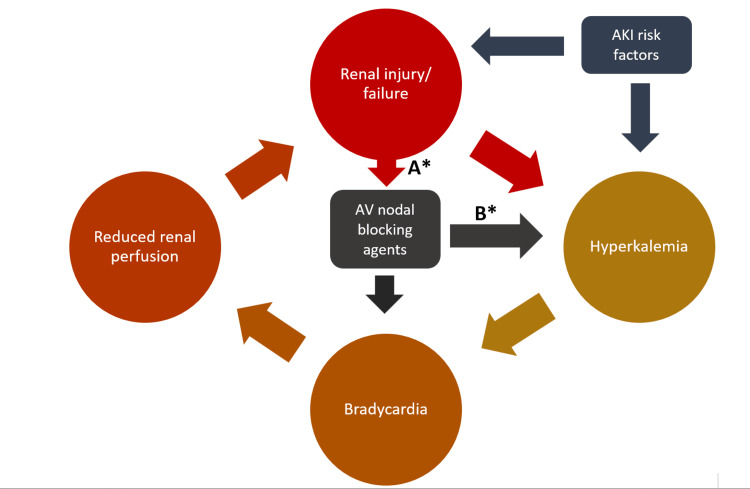
Pathophysiological vicious cycle of BRASH syndrome *Non-compulsory pathways that may be contributory to the cycle; A = poor clearance of renally cleared beta-blockers; B = non-selective beta-blockers that may cause hyperkalemia. AKI, acute kidney injury; AV, atrioventricular This figure was created by the authors of this article.

BRASH syndrome is a life-threatening condition that has straightforward management, especially if it is adequately and promptly identified at the initial presentation. Prior to the description of the syndrome in 2016 [1], some clinicians have managed it well without being aware of the condition or its pathophysiologic course. The syndrome is a sequela of the synergistic effect of AV nodal blockade and hyperkalemia [1,2]. In most cases, this AV nodal disease is a response to beta-blocking agents, such as the labetalol used in our patients, and the accompanying hyperkalemic effect in the syndrome is contributory even in mild hemodynamic derangements. Although the vicious cycle established by this synergy (Figure 4) is the cardinal pathophysiological explanation of the syndrome, there are still some gaps in the complete understanding of the syndrome and its management.

The syndrome is more common in older people who are on multiple medications for cardiovascular-related ailments [3], particularly, hypertension. Currently, there are limited epidemiological studies to ascertain any gender predilection. In a previous literature review of existing case reports [3], the review showed that 96% of the cases were all in patients between 51 and 97 years of age.

In patients on AV nodal blockers, BRASH syndrome is often precipitated by mild derangements from hypovolemic conditions such as gastrointestinal illnesses, poor oral intake, risk factors for kidney injury, or an up-titration of potassium-sparing anti-hypertensives, which increases potassium levels in the blood [2-6]. Our patient had preceding diarrhea, which triggered hypovolemia. Although severe hyperkalemia can cause bradycardia, it is unlike BRASH syndrome where even mildly elevated potassium levels can trigger the syndrome in the presence of AV nodal disease [1-3]. The bradycardic role of clonidine in our patient was not considered to be the cause. The patient has been on clonidine for a very long time, and the AV nodal-blocking effect of clonidine is not worse than beta-blockers. Beta-blockers have a stronger effect on AV blockade. However, we cannot rule out the synergistic effect of both medications.

The presenting symptoms of BRASH syndrome range from an asymptomatic state to a multi-organ dysfunction [2,3]. Most patients present anywhere within this spectrum with symptoms related to the precipitants. The symptoms of BRASH syndrome can mimic the presentation of cardiac disorders such as renal diseases, associated with cardiac conduction disorders [5-9]. Nevertheless, regardless of any presenting pattern, there is always profound bradycardia [2,3].

In making the diagnosis, besides the clinical history of the precipitants, BRASH syndrome is often clinically distinguished from other similar conditions by the presence of bradycardia amidst mild hyperkalemia, the absence of overdosing of AV nodal blockers, and an ECG with disproportionate bradycardia [1-3,7]. Bradycardia is typically seen in isolated hyperkalemia at serum potassium levels equal to or above 7.0 mEq/L, unlike in BRASH where bradycardia is seen in mild cases as low as 5.1 mEq/L [2,3]. Furthermore, typical ECG progression in isolated hyperkalemia is usually absent in BRASH syndrome, and this includes the progression from peaked T waves to flattening of P waves and PR prolongation and to QRS widening and bradycardia [2].

Beyond making a proper diagnosis of the syndrome, its management protocol remains a valid concern. As in our case, atropine was not sufficient to manage the bradycardia, and even the applied pacemaker was not utilized. Similar to other studies [1,3,7,8], transcutaneous pacemaker implantation is unnecessary in the management of the transient bradycardia that occurs in BRASH syndrome. Probably, the treatment of hyperkalemia appears to be the most effective factor. Furthermore, calcium gluconate or calcium chloride has the most immediate effect in reversing bradycardia caused by hyperkalemia [1,2].

AV-nodal blocking agents form the cornerstone for managing specific cardiovascular diseases; therefore, it is imperative to adopt a risk stratification system to identify patients with the greatest risk for BRASH syndrome and institute prompt supportive management whenever it occurs. Further literary works are required to increase knowledge of this syndrome. To prevent a recurrence, we reviewed our patient’s medication list and substituted it with a non-beta-blocking anti-hypertensive medication, Amlodipine, for the management of hypertension.

## Conclusions

BRASH syndrome is a life-threatening emergency that comprises a pentad of cardiorenal events and requires early identification and prompt treatment. A high index of suspicion for the syndrome is helpful in preventing the consequences that may result from its misdiagnosis. The avoidance of beta blockers and non-dihydropyridine calcium channel blockers in these patients cannot be over-emphasized and must be avoided in patients with a prior history of intrinsic cardiac conduction abnormalities of the heart.

## References

[1] Farkas J: PulmCrit (2022). PulmCrit - BRASH syndrome: bradycardia, renal failure, AV blocker, shock, hyperkalemia. https://emcrit.org/pulmcrit/brash-syndrome-bradycardia-renal-failure-av-blocker-shock-hyperkalemia/.

[2] Lizyness K, Dewald O (2022). BRASH syndrome. StatPearls [Internet].

[3] Farkas JD, Long B, Koyfman A, Menson K (2020). BRASH syndrome: bradycardia, renal failure, AV blockade, shock, and hyperkalemia. J Emerg Med.

[4] Newton E, Ransford G, Cipi A (2022). BRASH syndrome: a vicious cycle. https://www.emra.org/emresident/article/brash-syndrome/.

[5] Wong CK, Jaafar MJ (2021). Bradycardia, renal failure, atrioventricular nodal blockade, shock, and hyperkalemia: An important syndrome to recognize. Turk J Emerg Med.

[6] Sattar Y, Bareeqa SB, Rauf H, Ullah W, Alraies MC (2020). Bradycardia, renal failure, atrioventricular-nodal blocker, shock, and hyperkalemia syndrome diagnosis and literature review. Cureus.

[7] Khan A, Lahmar A, Ehtesham M, Riasat M, Haseeb M (2022). Bradycardia, renal failure, atrioventricular-nodal blockade, shock, and hyperkalemia syndrome: a case report. Cureus.

[8] Grigorov MV, Belur AD, Otero D, Chaudhary S, Grigorov E, Ghafghazi S (2020). The BRASH syndrome, a synergistic arrhythmia phenomenon. Proc (Bayl Univ Med Cent).

[9] Bailuni Neto JJ, Siqueira BL, Machado FC (2022). BRASH syndrome: a case report. Am J Case Rep.

